# Agricultural Management Practices to Sustain Crop Yields and Improve Soil and Environmental Qualities

**DOI:** 10.1100/tsw.2003.62

**Published:** 2003-08-20

**Authors:** Upendra M. Sainju, Wayne F. Whitehead, Bharat P. Singh

**Affiliations:** Agricultural Research Station, Fort Valley State University, Fort Valley, GA 31030, USA

**Keywords:** cover crop, crop yield, economic analysis, nitrogen fertilization, nitrogen fixation, nitrogen leaching, soil organic carbon, soil organic nitrogen, sustainable management practices, tillage practices

## Abstract

In the past several decades, agricultural management practices consisting of intensive tillage and high rate of fertilization to improve crop yields have resulted in the degradation of soil and environmental qualities by increasing erosion and nutrient leaching in the groundwater and releasing greenhouses gases, such as carbon dioxide (CO_2_) and nitrous oxide (N_2_O), that cause global warming in the atmosphere by oxidation of soil organic matter. Consequently, management practices that sustain crop yields and improve soil and environmental qualities are needed. This paper reviews the findings of the effects of tillage practices, cover crops, and nitrogen (N) fertilization rates on crop yields, soil organic carbon (C) and N concentrations, and nitrate (NO_3_)-N leaching from the soil. Studies indicate that conservation tillage, such as no-till or reduced till, can increase soil organic C and N concentrations at 0- to 20-cm depth by as much as 7–17% in 8 years compared with conventional tillage without significantly altering crop yields. Similarly, cover cropping and 80–180 kg N ha year fertilization can increase soil organic C and N concentrations by as much as 4–12% compared with no cover cropping or N fertilization by increasing plant biomass and amount of C and N inputs to the soil. Reduced till, cover cropping, and decreased rate of N fertilization can reduce soil N leaching compared with conventional till, no cover cropping, and full rate of N fertilization. Management practices consisting of combinations of conservation tillage, mixture of legume and nonlegume cover crops, and reduced rate of N fertilization have the potentials for sustaining crop yields, increasing soil C and N storage, and reducing soil N leaching, thereby helping to improve soil and water qualities. Economical and social analyses of such practices are needed to find whether they are cost effective and acceptable to the farmers.

### INTRODUCTION

Increasing crop production to feed the growing population was a major challenge to the agricultural community in the past few decades. As a result, management practices consisting of intensive tillage and high rate of fertilization were used to increase crop production. While these practices increased crop production by severalfold compared with less intensive tillage and no fertilization, little attention was paid to maintaining soil and environmental qualities. Tillage increased soil erosion and organic matter oxidation, thereby decreasing soil fertility and increasing soil and nutrient losses through runoff and leaching. This practice also caused the contamination of the surface and groundwater and increased the amount of sediments in streams and lakes.

The reduced organic matter content in soil decreased its quality by altering physical, chemical, and biological properties, such as water and nutrient retention capacities, aggregation, infiltration capacity, and microbial activities. Similarly, high rates of N and phosphorus (P) fertilization increased N leaching and P runoff from the soil surface, thereby increasing $NO_3$ and P concentrations in the surface and groundwater and consequently in eutrophication in the streams and lakes. This process increased the health hazard to humans and animals. Tillage and N fertilization also increased global warming by emitting $CO_2$ from soil organic matter oxidation and manufacturing of N fertilizer. Manufacturing a ton of N fertilizer can release 5 tons of C[1].

Adopting conservation tillage, cover cropping, and reduced rate of N fertilization can not only sustain crop yields but also improve soil and water qualities[2,3,4,5]. Soil quality is improved by increasing C and N storage in the soil and reducing soil erosion that can be obtained from using conservation tillage and cover cropping compared with conventional tillage and no cover croping[2,6,7]. This may help to reduce the concentration of $CO_2$ and N in the atmosphere. Similarly, water quality is improved when concentration of $NO_3$ is reduced, which can be done by reducing the rate of N fertilization. Conservation tillage reduces soil disturbance by limiting tillage to no-till where seeds are drilled and plowing is eliminated. Cover crops reduce soil exposure, use residual soil N thereby reducing N leaching[8,9], and may increase soil C and N storage by increasing C and N inputs through increased biomass production[4,5,10,11]. Similarly, N fertilization can increase soil organic C and N concentrations by increasing plant biomass production but can increase N leaching[3,12,13].

Carbon and N losses from soil due to tillage are contributory factors in global warming. Agriculture is responsible for 20% of global emissions of greenhouse gases[1]. Using improved agricultural management practices, over the next hundred years, soils could restore about 7–12% of global fossil fuel emissions at 1990 levels through C and N sequestration potentials[1].

### CROP YIELDS

#### Tillage

Crop yields produced by using conservation tillage have been reported to be similar or higher than those produced by using conventional tillage. Yields of corn (*Zea mays* L.)[8,14,15], cotton (*Gossypium hirsutum* L.) lint[16,17,18], wheat (*Triticum aestivum* L.)[19,20,21], and sorghum (*Sorghum bicolor* L.)[19] using conservation tillage have been found to be not significantly different from using conventional tillage. Sainju et al.[22] found that silage corn yield was not significantly different between tillage practices, but cotton lint yield was greater with no-tillage (NT) than with moldboard plowing (MP) (conventional tillage), whereas sorghum grain yield was greater with chisel plowing (CP) (reduced tillage) and MP than with NT (Table 1). Similarly, tomato (*Lycopersicum esculentum* Mill.) yield was not significantly different with tillage treatments in 1997, but was greater with CP and MP than with NT in 1996. The results suggests that no-tillage may be as good as conventional tillage in producing yields of cereal crops but reduced tillage, such as CP, may be needed to sustain yields of vegetable crops, such as tomato. Plant residues at the soil surface in conservation tillage systems have been reported to delay the germination and growth of crops compared with conventional tillage, but the final crop yields have been reported to be not influenced by tillage systems[8,16,21].

**TABLE 1**
**Effects of Tillage, Cover Crop, and N Fertilization Rates on Yields of Fresh-Market Tomato, Silage Corn (Dry Matter Weight), Cotton Lint, and Sorghum Grain[22]**

| | Tomato ($Mg\ ha^{-1}$) | | Silage Corn ($Mg\ ha^{-1}$) | | Cotton Lint ($kg\ ha^{-1}$) | Sorghum Grain ($Mg\ ha^{-1}$) |
|---|---|---|---|---|---|---|
| **Treatment** | **1996** | **1997** | **1998** | **1999** | **2000** | **2001** |
| Tillage[1] | | | | | | |
| NT | 35.0b[2] | 32.1a | 14.5a | 20.3a | 890a | 2.2b |
| CP | 66.4a | 33.5a | 14.6a | 21.5a | 708ab | 3.4a |
| MP | 62.9a | 30.5a | 15.7a | 21.1a | 595b | 3.9a |
| Cover crop[3] | | | | | | |
| HV | 56.7a | 34.0a | 14.8a | 24.6a | 660a | 3.5a |
| NHV | 53.9a | 33.4a | 15.0a | 17.3b | 699a | 2.8b |
| N fertilization rate ($kg\ N\ ha^{-1}$) | | | | | | |
| 0 | 49.5b | 26.6b | 15.5a | 21.6a | 673b | 2.8b |
| 60–90 | 58.1a | 36.0a | 14.9a | 21.1a | 783a | 3.1b |
| 120–180 | 56.6a | 33.6a | 14.4a | 20.2a | 736ab | 3.7a |

1 Tillage are NT, no-till; CP, chisel plowing; and MP, moldboard plowing.
2 Within a column and a set, numbers followed by different letter are significantly different ($p <$ 0.05, least significant difference test).
3 Cover crops are HV, hairy vetch; and NHV, no hairy vetch.

### Cover Crops

Cover crops, primarily legumes, can increase crop yields compared with nonlegumes or no cover crops. Sainju et al.[22] observed that legume cover crops, such as hairy vetch (*Vicia villosa* Roth) and crimson clover (*Trifolium incarnatum* L.), increased yields of silage corn, sorghum grain, tomato, eggplant (*Solanum melogena* L.), and bell pepper (*Capsicum annuum* L.) compared with nonlegume cover crops, such as rye (*Secale cereale* L.), or no cover crop (Tables 1 and 2). They observed that yield increases with hairy vetch and crimson clover were similar to those obtained with fertilization of 60–180 kg N $ha^{-1}$. Similar increased yields of corn, cotton, sorghum, and tomato with legume cover crops have been reported by researchers in various regions of the U.S. (Table 3)[23]. The yield increases of summer crops following legume cover crops were equivalent to those produced by fertilization of 15–200 kg N $ha^{-1}$[23]. Nonlegume cover crops, however, produced crop yields similar to or less than those did without a cover crop.

**TABLE 2**
**Effects of Cover Crops and N Fertilization Rates on Yields of Fresh-Market Tomato, Eggplant, and Bell Pepper[22]**

| | Tomato ($Mg\ ha^{-1}$) | | | Eggplant ($Mg\ ha^{-1}$) | Bell Pepper ($Mg\ ha^{-1}$) |
|---|---|---|---|---|---|
| **Treatment** | **1996** | **1997** | **1999** | **2000** | **2001** |
| Rye | 19.0b[1] | 13.6c | 37.0c | 21.0c | 6.3b |
| Hairy vetch | 40.2a | 31.5a | 75.2a | 52.1a | 34.2a |
| Crimson clover | 40.9a | 30.0a | 65.4ab | 45.5a | 29.1a |
| Control[2] | 20.0b | 17.3bc | 56.1b | 23.7c | 7.6b |
| HN[2] | 39.1a | 27.9ab | 66.2ab | 44.3ab | 28.8a |
| FN[2] | 43.1a | 27.0ab | 67.4ab | 37.1b | 30.1a |

1 Within a column, numbers followed by different letter are significantly different ($p < 0.05$, least significant difference test).
2 Control contains no cover crop or N fertilization; HN denotes the half N rate for tomato, eggplant, and bell pepper (80–90 kg N $ha^{-1}$); and FN is the full N rate for tomato, eggplant, and bell pepper (160–180 kg N $ha^{-1}$).

**TABLE 3**
**Cover Crop Yields and Their N Contributions, and Yield and N Uptake of Succeeding Crop[23]**

| | | | Cover Crop | | Succeeding Crop | |
|---|---|---|---|---|---|---|
| **Reference and Location** | **Cover Crop** | **Succeeding Crop/Rotation** | **Yield ($Mg\ ha^{-1}$)** | **N Contribution ($kg\ ha^{-1}$)** | **Yield ($Mg\ ha^{-1}$)** | **N Uptake ($kg\ ha^{-1}$)** |
| Decker et al.[24], Maryland | Hairy vetch | Field corn | 2.9–5.1 | 109–206 | 7.2–8.9 | 140–204 |
| | Austrian winter pea | | 1.9–4.7 | 73–180 | 7.5–8.9 | 144–201 |
| | Crimson clover | | 2.1–6.5 | 59–170 | 7.2–8.9 | 138–190 |
| | Wheat | | 2.1–4.0 | 35–42 | 6.2–7.2 | 128–165 |
| | Control | | — | — | 6.3–7.7 | 121–175 |
| Ebelhar et al.[25], Kentucky | Hairy vetch | Field corn | 5.1 | 209 | 6.4 | — |
| | Big flower vetch | | 1.9 | 60 | 4.2 | — |
| | Crimson clover | | 2.4 | 56 | 4.4 | — |
| | Rye | | 3.4 | 36 | — | — |
| | Fallow | | — | — | 4.4 | — |

**TABLE 3 (continued)**
**Cover Crop Yields and Their N Contributions, and Yield and N Uptake of Succeeding Crop[23]**

| | | | Cover Crop | | Succeeding Crop | |
|---|---|---|---|---|---|---|
| Reference and Location | Cover Crop | Succeeding Crop/Rotation | Yield (Mg ha$^{-1}$) | N Contribution (kg ha$^{-1}$) | Yield (Mg ha$^{-1}$) | N Uptake (kg ha$^{-1}$) |
| Kamprath et al.[26], North Carolina | Austrian winter pea | Field corn | 2.0 | 51 | 3.0 | — |
| | Oat + hairy vetch | | 2.9 | 87 | 3.3 | — |
| | Hairy vetch | | 3.0 | 120 | 3.9 | — |
| | Austrian winter pea | Cotton | 2.1 | 79 | 95.9 | — |
| | Oat + hairy vetch | | 3.3 | 106 | 87.7 | — |
| | Hairy vetch | | 3.2 | 131 | 87.2 | — |
| Kelly et al.[27], Maryland | Hairy vetch mulch | Tomato | — | — | 104 | — |
| | Polyethylene mulch | | — | — | 80 | — |
| | Bare soil | | — | — | 55 | — |
| Kuo et al.[28], Washington | Rye | Silage corn | 5.3 | 60 | 7.4 | 112 |
| | Annual ryegrass | | 7.1 | 56 | 7.2 | 62 |
| | Hairy vetch | | 3.2 | 120 | 12.3 | 179 |
| | Austrian winter pea | | 3.9 | 100 | 9.6 | 118 |
| | Canola | | 3.3 | 44 | 7.8 | 78 |
| | Control | | 2.1 | 30 | 7.6 | 73 |
| Hargrove[10], Georgia | Rye | Sorghum | 4.0 | 38 | 2.6 | — |
| | Crimson clover | | 7.2 | 170 | 3.9 | — |
| | Subterranean clover | | 4.0 | 114 | 3.8 | — |
| | Hairy vetch | | 4.3 | 153 | 4.0 | — |
| | Common vetch | | 4.3 | 134 | 3.7 | — |
| Touchton et al.[29], Alabama | Fallow | Cotton | 2.7 | 31 | 0.6 | — |
| | Crimson clover | | 4.5 | 95 | 0.9 | — |
| | Common vetch | | 4.9 | 118 | 0.8 | — |

Legume cover crops increase crop yields because of greater biomass yields and N concentration than nonlegume or no cover crops (Table 3)[23]. While N supplied by legumes ranged from 51–209 kg $ha^{-1}$, nonlegumes supplied 35–60 kg N $ha^{-1}$. Because of lower C:N ratio, residues of legume cover crops decompose rapidly in the soil[30,31] and release N in the soil greater and earlier than do nonlegumes (Fig. 1)[5,32]. Because the half-life of cover crop N ranges from 2–9 weeks[5,33,34], N released by legume cover crops is usually synchronized with N needs of summer crops[33,35]. As a result, N uptake by crops following legume cover crops is usually higher than following nonlegume or no cover crops (Table 3).

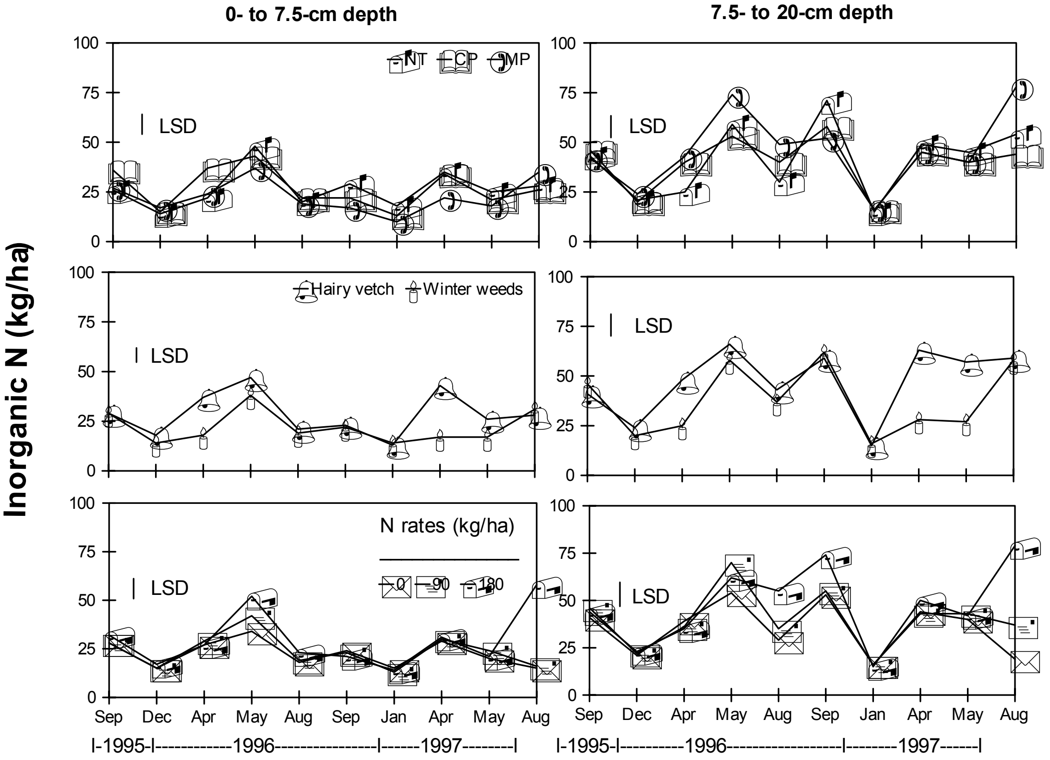

**FIGURE 1.** Soil inorganic ($NH_4$ + $NO_3$) content at 0- to 7.5- and 7.5- to 20.0-cm depths from September 1995 to August 1997 as influenced by tillage, cover crops, and N fertilization rates. NT denotes no-till; CP, chisel plowing; and MP, moldboard plowing. LSD is the least significant difference at $p < 0.05$[36].

### Nitrogen Fertilization

Crop yields usually increase with N fertilization. Increasing the N rate, however, does not proportionally increase yields. Yields are increased to a maximum level at a N rate depending on the soil, climate, and environmental conditions, after which they decrease with further increase in N rate. As a result, accumulation of soil residual N after crop harvest and the potential for N leaching increase. A high N rate increases plant biomass yield at the expense of grain or fruit yield. Sainju et al.[22] found that yields of tomato, silage corn, cotton lint, eggplant, and bell pepper were not significantly different between N rates of 60–90 and 120–180 kg $ha^{-1}$ (Tables 1 and 2). Therefore, a high N rate to produce crop yields should be avoided to reduce the potential for N leaching and the degradation of water quality and to decrease the cost of fertilization if low N rate can produce similar yields. To obtain a N rate that sustains crop yield and reduces N leaching, periodic N analysis of soil and plant samples should be conducted.

### SOIL ORGANIC CARBON AND NITROGEN AND PHYSICAL PROPERTIES

Organic C and N concentrations in the soil measure organic matter status, which influences soil quality and productivity due to its favorable effects on physical, chemical, and biological properties[37,38]. Soil organic C and N play critical roles in nutrient cycling, water retention, root growth, plant productivity, and environmental quality. Organic C and N concentrations in the soil can be influenced by management practices, such as tillage, cover cropping, and N fertilization[2,4,5,39].

#### Tillage

Tillage enhances mineralization of soil organic C and N by incorporating plant residues, disrupting soil aggregates, increasing aeration, and altering soil temperature and moisture that favor microbial degradation[40,41,42]. The mineralization will continue until organic C and N lost by tillage are replaced by the addition of plant residues or soil amendments[43,44]. The rate of declining of soil organic C and N due to cultivation depends on the types of crop grown and crop rotation[45], method of disposal of crop residue[46], soil characteristics[47], fertilization[48], and tillage practices[42]. Fallowing the land without plant residues returned to the soil also increases the rate of depletion of organic C and N[7].

Conservation tillage increases soil organic C and N concentrations compared with conventional tillage by reducing oxidation of organic matter and aggregate degradation[2,6,7]. Placement of plant residue at the soil surface in NT reduces its contact with soil microorganisms for decomposition, thereby increasing the concentrations of organic C and N with NT compared with MP[6,22,39]. In an experiment on the effects of tillage, cover crop, and N fertilization on soil organic C and N levels in Georgia (U.S.), Sainju et al.[22] reported that soil organic C and N levels at 0- to 20-cm depth were greater with NT than with MP from 1995–2001 (Figs. 2A and 3A). The levels with CP were in between those with NT and MP. Although the levels decreased from 1994–2001 in all tillage systems, the rate of decline was greater with MP than with NT and CP. As a result, they observed that, from 1994–2001, soil organic C and N contents decreased by 8–16% with NT and 15–25% with CP and MP.

The transition of conventional tillage to NT in the beginning can result in incomplete amelioration of compacted soil over the winter, thereby increasing soil bulk density and reducing root growth and crop yield[49,50,51]. As NT is continued over years, crop yields can be similar to or greater with NT than with conventional tillage[16,49]. The use of heavy machinery in conventional tillage can also result in soil compaction, thereby reducing crop root growth and yield[16,52,53].

#### Cover Crops

Cover crops grown in the winter without the application of fertilizers, herbicides, and pesticides after the harvest of the summer crops fix atmospheric $CO_2$ and increase organic C level in the soil compared with bare fallow[4,10,51]. Because cover crops use residual fertilizer and soil nutrients, such as N, and assimilate in the plant biomass, they also recycle soil inorganic N and increase organic N level at the surface soil[5,10]. If cover crops are legumes, they also fix atmospheric N and enrich both organic and inorganic N levels in the soil that help to increase crop production[10,11,32]. The extent of increase of organic C and N following incorporation of cover crop residues depends on a number of factors, including the amount and quality of residues, rate and manner of application, soil type, frequency of tillage, and climatic condition[54,55].

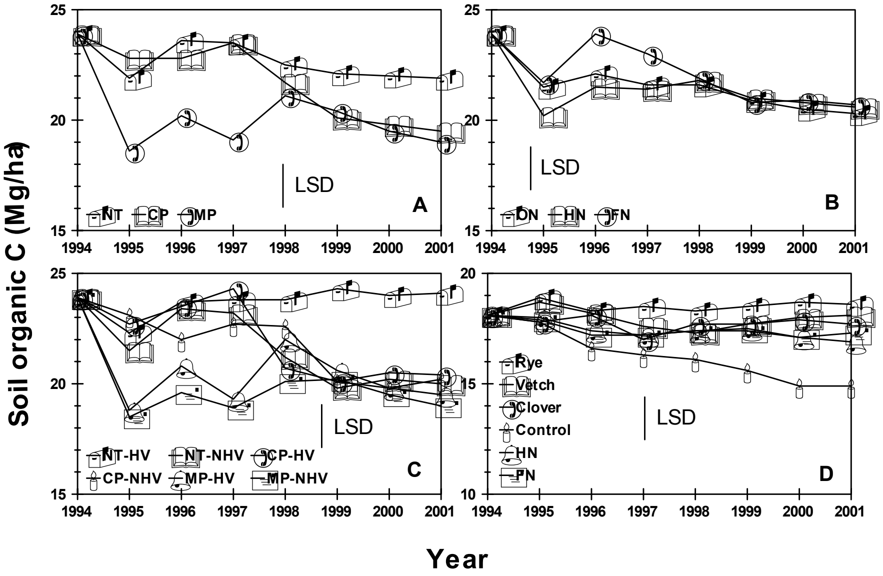

**FIGURE 2**. Soil organic C concentration as influenced by tillage, cover crops, and N fertilization rates. (A, B, and C) NT denotes no-till; CP, chisel plowing; MP, moldboard plowing; HV, hairy vetch; NHV, no hairy vetch; ON, 0 kg N/ha; HN, 60–90 kg N/ha; and FN, 120–180 kg N/ha. (D) Vetch denotes hairy vetch; clover, crimson clover; control, no cover crop or N rate; HN, 80–90 kg N/ha; and FN, 160–180 kg N/ha. LSD denotes least significant difference at $p < 0.05$[22].

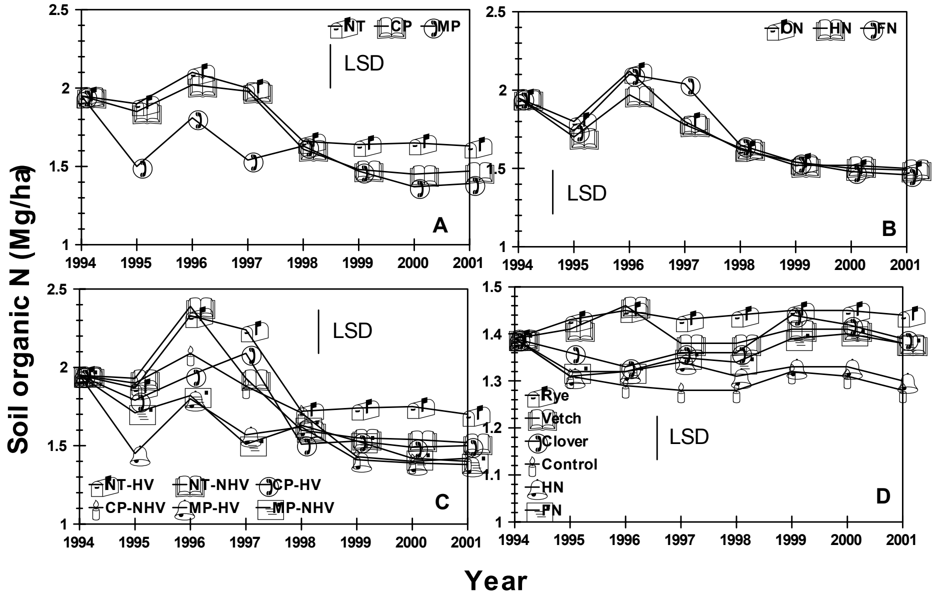

**FIGURE 3**. Soil organic N concentration as influenced by tillage, cover crops, and N fertilization rates. (A, B, and C) NT denotes no-till; CP, chisel plowing; MP, moldboard plowing; HV, hairy vetch; NHV, no hairy vetch; ON, 0 kg N/ha; HN, 60–90 kg N/ha; and FN, 120–180 kg N/ ha. (D) Vetch denotes hairy vetch; clover, crimson clover; control, no cover crop or N rate; HN, 80–90 kg N/ha; and FN, 160–180 kg N/ha. LSD denotes least significant difference at $p < 0.05$[22].

Another factor that influences soil organic C and N levels is the rate at which cover crop residue decomposes[4,5,42]. The balance between the amount of residue added to the soil and the rate at which it decomposes determines the levels of soil organic C and N[56,57]. The decomposition rate varies with the species and growth of cover crop at incorporation[58] due to variation in chemical composition, such as N and lignin concentrations and C:N ratio[59,60]. The decomposition rate of plant residues depends on the size of available C and N pools which affect the size of microbial biomass[61]. Climate, particularly temperature and moisture, also regulates the rate of decomposition[62].

The effectiveness of legume and nonlegume cover crops in increasing the levels of organic C and N is mixed (Table 4). While some researchers[10,11,63,64] have reported that legumes, such as vetch and clover, are more effective in enriching organic C and N, others[4,5,51] have observed that nonlegumes, such as rye and ryegrass, are more effective because of greater biomass yield and higher C:N ratio which reduces the rate of decomposition. Sainju et al.[22] observed that rye maintained greater soil organic C and N levels than hairy vetch and crimson clover from 1994–2001 (Figs. 2D and 3D). As a result, they observed that, from 1994–2001, organic C and N contents increased by 3–4% with rye but decreased by 1% with hairy vetch and crimson clover.

**TABLE 4**
**Cover Crop Effects on Soil Organic C and N Concentrations[23]**

| Reference | Cover Crop | Soil Depth (cm) | Organic Component | |
|---|---|---|---|---|
| | | | C (g $kg^{-1}$) | N (g $kg^{-1}$) |
| Frye et al.[63] | Fallow | 0–7.5 | 10.6 | 1.2 |
| | Hairy vetch | | 13.5 | 1.5 |
| | Big flower vetch | | 12.7 | 1.4 |
| | Rye | | 11.5 | 1.2 |
| Hargrove[10] | Initial | 0–7.5 | 11.3 | 0.77 |
| | Fallow | | 7.9 | 0.58 |
| | Rye | | 8.7 | 0.65 |
| | Crimson clover | | 8.4 | 0.65 |
| | Subterranean clover | | 10.0 | 0.81 |
| | Hairy vetch | | 9.7 | 0.80 |
| | Common vetch | | 10.2 | 0.63 |
| | Initial | 7.5–15 | 6.1 | 0.49 |
| | Fallow | | 4.8 | 0.37 |
| | Rye | | 5.4 | 0.42 |
| | Crimson clover | | 4.9 | 0.41 |
| | Subterranean clover | | 5.5 | 0.48 |
| | Hairy vetch | | 5.5 | 0.51 |
| | Common vetch | | 5.1 | 0.45 |
| Kuo et al.[28] | Fallow | 0–15 | 15.7 | 1.22 |
| | Austrian winter pea | | 16.0 | 1.26 |
| | Hairy vetch | | 15.8 | 1.28 |
| | Canola | | 15.4 | 1.23 |
| | Cereal rye | | 16.6 | 1.34 |
| | Annual ryegrass | | 16.6 | 1.28 |

**TABLE 4 (continued)**
**Cover Crop Effects on Soil Organic C and N Concentrations[23]**

| Reference | Cover Crop | Soil Depth (cm) | Organic Component | |
|---|---|---|---|---|
| | | | C (g $kg^{-1}$) | N (g $kg^{-1}$) |
| McVay et al.[11] | Fallow | 0–5 | 8.5-10.1 | 1.0-1.3 |
| | Wheat | | 8.9-11.8 | 1.1-1.4 |
| | Crimson clover | | 10.6-12.8 | 1.3-1.5 |
| | Hairy vetch | | 10.2-11.8 | 1.3-1.5 |
| | Fallow | 5–10 | 7.2-8.7 | 0.9-1.0 |
| | Wheat | | 7.3-9.5 | 1.0-1.2 |
| | Crimson clover | | 7.7-10.3 | 1.0-1.2 |
| | Hairy vetch | | 7.4-9.3 | 1.0-1.2 |
| Touchton et al.[29] | Fallow | 0–11 | 7.0 | 0.32 |
| | Crimson clover | | 8.7 | 0.43 |
| | Hairy vetch | | 10.8 | 0.42 |
| Wilson et al.[64] | Initial | 0–15 | 17.0 | 1.6 |
| | Fallow | | 12.0 | 1.2 |
| | Grasses | | 15.0 | 1.8 |
| | Legumes | | 16.0 | 2.0 |

Although nonlegumes have lower N concentration than legumes, greater soil organic N level with nonlegumes may have resulted from increased biomass production[4,51] and close association of soil organic C and with organic N[57,65,66]. The difference in soil and climatic conditions among regions probably has influenced the growth and production of cover crop biomass that may have influenced the organic C and N levels in the soil at various regions in the U.S. (Table 4).

While tillage and cover cropping interact each other on the levels of soil organic C and N, a combination of conservation tillage and cover cropping may be more effective than either practice alone to increase the C and N levels. Sainju et al.[22] observed that soil organic C and N levels were greater in NT with hairy vetch than in NT alone or in CP and MP with or without hairy vetch (Figs. 2A, 2C, 3A, and 3C). This may be due to the difference in the amount and placement of plant residue between the treatments. Cover crops have usually greater biomass yield than weeds in the bare fallow[22]. When the biomass is placed at the soil surface in NT, reduced contact of the residue with soil microorganisms decreases its decomposition compared with that incorporated into the soil in conventional tillage, thereby maintaining greater levels of organic C and N in NT[2,22,39].

Cover crops also influence soil physical properties, such as water retention, aggregation, infiltration capacity, bulk density, temperature, and erosion. Smith et al.[66] suggested that cover crops improve soil water retention by reducing evaporation due to mulch effect, increasing soil infiltration capacity, retaining precipitation, and reducing water loss from the cover crop canopy. Roberson et al.[67] observed that cover cropping improved soil aggregation by increasing slaking resistance and saturated hydraulic conductivity compared with no cover cropping. Similarly, several researchers[11,68,69] found that cover cropping increased the proportion of water stable aggregates and infiltration capacity of the soil. Cover crop mulch can reduce soil temperature, thereby promoting root development of the succeeding crops[27,32,70]. Frye et al.[63] observed that cover crop reduced soil erosion from 18–2 Mg $ha^{-1}$ $year^{-1}$. Similarly, Langdale et al.[71] found that cover crops reduced soil erosion from 47–96% in the southeastern U.S.

### Nitrogen Fertilization

Nitrogen fertilization can increase plant biomass production, thereby increasing soil organic C and N levels compared with no fertilization[3,12,13]. Sainju et al.[22] observed that soil organic C and N levels increased with 120–180 kg N $ha^{-1}$ compared with 0 kg N $ha^{-1}$ (Figs. 2B, 2D, 3B, and 3D). As a result, they observed that, from 1994–2001, organic C and N levels decreased by 2–6% with N fertilization compared with 6–18% decrease with no N fertilization. The increased levels of organic C and N with N fertilization were, however, less than the increases obtained by using conservation tillage and cover crops. Sainju et al.[36,51] found that N fertilization immediately increased labile pools of soil C, such as potential C mineralization, indicating increased microbial activities.

## SOIL NITRATE-NITROGEN MOVEMENT AND LEACHING

Nitrate-N is soluble in water and also is the form of N taken up by plants. Because it carries a negative charge, it is not retained by negatively charged soil colloids[72,73]. As a result, it is free to move with percolating water within the soil profile, which results in $NO_3$ leaching from the soil and contamination of groundwater[8]. Nitrate-N concentration in excess of 10 mg $l^{-1}$ in the drinking water has been considered a health hazard to humans and animals[74,75].

When amendments, such as manure, crop residue, or N fertilizer, are applied to the soil to supply N requirement of the crop, only a part of it is taken up by plants. The remaining N accumulates as soil residual N that, if not immobilized in the soil, is subject to leaching and denitrification. In humid regions, $NO_3$ leaching occurs mostly during autumn, winter, and spring seasons when evapotranspiration is low and precipitation exceeds the water-holding capacity of the soil[8,76]. The accumulated amount of residual N and its movement and leaching in the soil profile depend on soil properties, climatic condition, plant N uptake, N transformation, evapotranspiration, drainage, and management practices[77,78,79]. Although N contamination of groundwater results from several sources, such as industrial wastes, municipal landfills, mining, or septic systems, agricultural practices remain a major source[80,81,82]. About 15–55% of N applied to crops can be lost by leaching every year[74,83].

### Tillage

Tillage can enhance the mineralization of organic N from crop residue and soil and increase the accumulation of residual $NO_3$-N[72,83,84]. Sainju et al.[85] observed that, although $NO_3$ accumulation in the soil at 0- to 120-cm depth in the fall (autumn) after 3 years was not influenced by tillage, accumulation in the following spring was greater with CP and MP than with NT (Table 5). The total amount of $NO_3$-N lost from fall to spring was, however, more than doubled with NT than with CP and MP. These results were similar to those observed by various researchers[86,87,88] who have suggested that because of the presence of large macro-pores, $NO_3$ leaching loss increased more with NT than with conventional tillage.

### Cover Crops

Cover crops can reduce N leaching from the soil profile by using residual N left after crop harvest in the autumn[8,9]. Cover crops also use soil water that can solubilize N and transport it through runoff and leaching. The effectiveness of cover crops to reduce N leaching, however, differs among species. Cover crop species that establish quickly and grow vigorously in the autumn are

**TABLE 5**
**Nitrate-N Content at 0- to 60-, 60- to120-, and 0- to 120-cm Depth in the Fall (September 1997) and the Spring (March 1998) as Influenced by 3 Years of Tillage, Cover Cropping, and N Fertilization[85]**

| Treatment | 0- to 60-cm Depth | | | 60- to 120-cm Depth | | | 0- to 120-cm Depth | | |
|---|---|---|---|---|---|---|---|---|---|
| | Fall | Spring | Loss | Fall | Spring | Loss | Fall | Spring | Loss |
| | ($kg\ ha^{-1}$) | | | | | | | | |
| Tillage[1] | | | | | | | | | |
| NT | 95a[2] | 25b | 70 (74)[3] | 127a | 68b | 59 (46)[3] | 222a | 93b | 129 (58)[3] |
| CP | 68b | 26b | 42 (62) | 147a | 134a | 12 (9) | 215a | 160a | 55 (26) |
| MP | 107a | 58a | 49 (46) | 144a | 136a | 7 (5) | 250a | 194a | 56 (22) |
| Cover crop[4] | | | | | | | | | |
| HV | 104a | 43a | 61 (58) | 170a | 128a | 42 (25) | 274a | 171a | 103 (38) |
| NHV | 76b | 30b | 47 (61) | 107b | 98b | 10 (9.0) | 184b | 127b | 56 (34) |
| N fertilization, $kg\ ha^{-1}$ | | | | | | | | | |
| 0 | 51b | 26b | 26 (50) | 76b | 65b | 11 (15) | 127c | 91b | 37 (29) |
| 90 | 83b | 29b | 54 (65) | 160a | 113ab | 48 (30) | 243b | 142b | 104 (42) |
| 180 | 136a | 55a | 82 (60) | 180a | 161a | 19 (11) | 316a | 216a | 101 (32) |

1 Tillage are NT, no-till; CP, chisel plowing; and MP, moldboard plowing.
2 Within a column and a set, numbers followed by different letter are significantly different ($p < 0.05$, least significant difference test).
3 Number in parenthesis denote % decrease in soil $NO_3$ content from fall to spring.
4 Cover crops are HW, hairy vetch; and NHV, no hairy vetch.

most effective in reducing N leaching[70]. Kuo et al.[28] reported that nonlegume cover crops, such as rye and annual ryegrass, removed $NO_3$ from the soil and reduced its leaching more effectively than legume cover crops, such as hairy vetch, or the control without a cover crop. They noticed that rye and annual ryegrass established well early in the autumn and produced extensive root systems for scavenging soil residual $NO_3$ compared with hairy vetch. Similarly, McCracken et al.[89] observed that rye reduced $NO_3$ concentration in the soil leachate by 94% compared with 48% by hairy vetch. The effectiveness of hairy vetch in reducing $NO_3$ leaching was mostly limited during spring when it grew vigorously. In a review of literature, Sainju and Singh[23] noted that nonlegume cover crops reduced $NO_3$ leaching by 29–94% compared with -6–48% by legume cover crops (Table 6). In contrast, Sainju et al.[85] found that hairy vetch, because of higher N concentration, increased soil residual N in the autumn and its loss from autumn to the following spring compared with no hairy vetch (Table 5). Similarly Kuo et al.[28] observed that hairy vetch increased $NO_3$ leaching compared with the control without a cover crop. Campbell et al.[77] found that fallowing followed by legume cover cropping increased $NO_3$ leaching from the soil. Nitrogen leaching following legume cover cropping, however, can be lower than following N fertilization due to slower N release from legumes than from fertilizer[77,90,91].

### Nitrogen Fertilization

Nitrogen fertilization increases N leaching because of the rapid release of N in the soil due to its solubility in water and inefficiency in crop N uptake. Crops are unable to use 100% of the applied

**TABLE 6**
**Reduction in $NO_3^-$ Leaching from Soil due to Cover Crops[23]**

| Reference | Cover Crop | Reduction Due to Cover Crop (%) |
|---|---|---|
| Bertilsson[92] | Rape | 62 |
| Chapman et al.[93] | Sweet clover | 1 |
| | Purple vetch | 10 |
| | Mustard | 80 |
| Karraker et al.[94] | Rye | 72 |
| McCracken et al.[89] | Rye | 94 |
| | Hairy vetch | 48 |
| Meisinger et al.[95] | Rye | 29 |
| | Hairy vetch | -6 |
| Morgan et al.[96] | Oat | 48 |
| | Rye | 62 |
| | Timothy | 33 |
| Volk and Bell[97] | Turnip | 84 |

N. Nitrogen recovery seldom exceeds 70% of the applied N and averages 50% for most crops[74,98,99]. As a result, a portion of N applied to crops remains as residual N in the soil that is subjected to leaching. As the rate of N fertilization increases, the potential for N leaching also increases[100,101]. Lowrance and Smittle[102] found that 290 out of 388 kg N $ha^{-1}$ applied to a vegetable-cereal production system was available as residual N for leaching at the end of harvest. Similarly, Sainju et al.[85] observed that soil residual N in the autumn after tomato harvest and its loss from autumn to spring increased with increasing rate of N fertilization (Table 5, Fig. 4). Liang et al.[103] and Liang and McKenzie[104] found that change in soil $NO_3$ level between autumn and spring was a function of autumn $NO_3$ level and overwinter precipitation. A direct linear relationship exists between the rate of N fertilization and amount of soil residual N available for leaching[100,101].

### SUSTAINABLE MANAGEMENT PRACTICES

Management practices that produce optimum crop yields, increase soil organic C and N concentrations, and reduce N leaching from the soil are needed for sustaining crop yields and improving soil, water, and environmental qualities. The practices chosen, however, may vary with the types of crops grown and the soil. For example, Sainju et al.[22] observed that reduced tillage, such as CP, produced tomato fruit and sorghum grain yields similar to that produced by conventional tillage, such as MP (Table 1). In contrast, silage corn and cotton lint yields were similar to or greater with NT than with CP and MP. Soil organic C and N concentrations were greater with NT than with CP and MP, regardless of crop types (Figs. 2A and 3A). Similarly, $NO_3$-N lost from the soil at 0- to 120-cm depth from autumn to spring were greater with NT than with CP and MP, indicating greater N leaching with NT (Table 5).

With regard to the cover crops, legumes can increase crop yields better than do nonlegumes or no cover crops (Tables 1, 2, and 3). Legumes can also increase crop yields similar to those did by N fertilization. Legumes, however, may not be as effective as nonlegumes in increasing soil organic C and N concentrations or reducing N leaching from the soil, although they are more

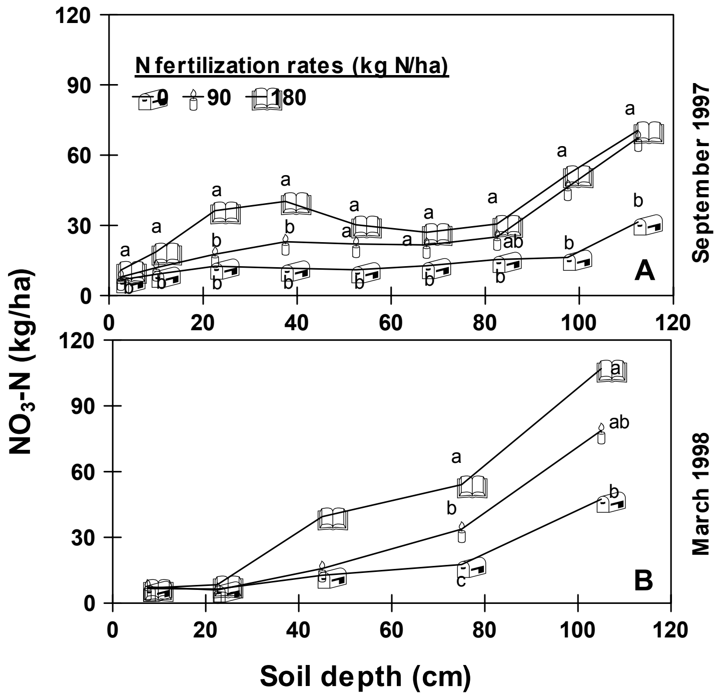

**FIGURE 4.** Soil $NO_3$-N content with depth in autumn (September 1997) and spring (March 1998) as influenced by N fertilization rates. Nitrate content at a particular depth is plotted at the midpoint of the depth range. Symbols followed by different letter at a particular depth are significantly different at $p < 0.05$ by the least square means test[85].

effective than no cover crops (Figs. 2D and 3D, Table 6). In contrast, nonlegumes do not increase crop yields compared with no cover crops, but they can increase soil organic C and N concentrations and reduce N leaching.

A mixture of legumes and nonlegumes may, however, produce crop yields similar to that produced by legumes and N fertilization and improve soil and water qualities similar to that improved by nonlegumes. Although evidence of similar corn and cotton yields produced by hairy vetch and a mixture of oat and hairy vetch is present (Table 3)[105], further research is necessary to find if a combination of legume and nonlegume cover crops can sustain crop yields and improve soil and water qualities.

Nitrogen fertilization can increase crop yields and soil organic C and N concentrations, but a higher rate of fertilization that is more than required by crops can increase N leaching. Studies have shown that reducing the rate of N fertilization by half can produce crop yields (Tables 1 and 2) and improve soil organic C and N concentrations (Figs. 2B and 3B) similar to those done by the recommended rate. Therefore, reducing the rate of N fertilization not only can sustain crop yields and maintain soil and water qualities, but can also reduce the cost of fertilization. Periodic analysis of soil and plant samples, however, may be needed to determine the exact rate of N fertilization for sustaining crop yields, maintaining soil quality, and reducing potential for N leaching.

Based on these results, a combination of management practices consisting of conservation tillage systems, such as NT and CP, mixture of legume and nonlegume cover crops, and reduced rate of N fertilization may be used to sustain crop yields and improve soil and water qualities. For example, studies have shown that combining conservation tillage with legume cover crop produced similar crop yields or soil $NO_3$ accumulation and movement, but increased soil organic C and N concentrations better than conservation tillage or cover cropping alone (Tables 1 and 5, Fig. 2). Similarly, cotton lint yield increased by combining NT with rye or rye and hairy vetch mixture, or with 60 kg N ha$^{-1}$ compared with NT alone (Figs. 5 and 6).

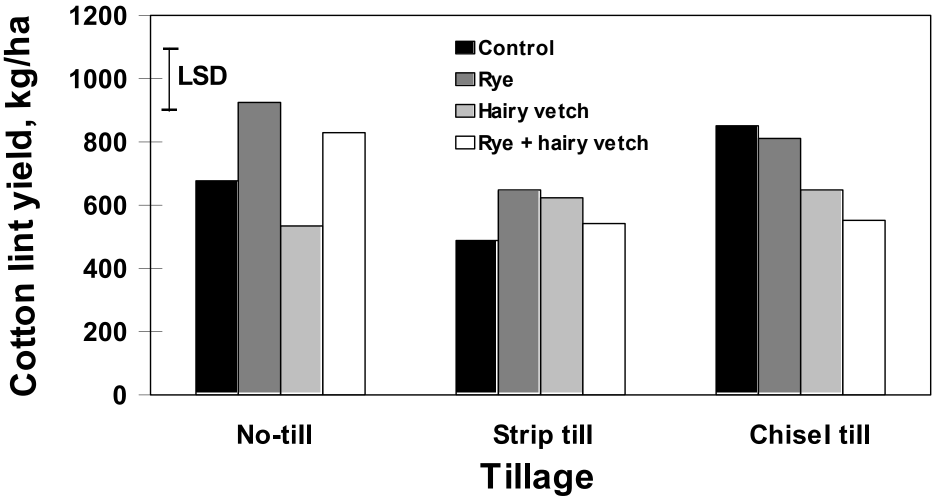

**FIGURE 5.** Cotton lint yield as influenced by tillage and cover crops averaged across N fertilization rates (0, 60, and 120 kg N/ha) and years (2000 and 2002). LSD denotes least significant difference at $p < 0.05$.

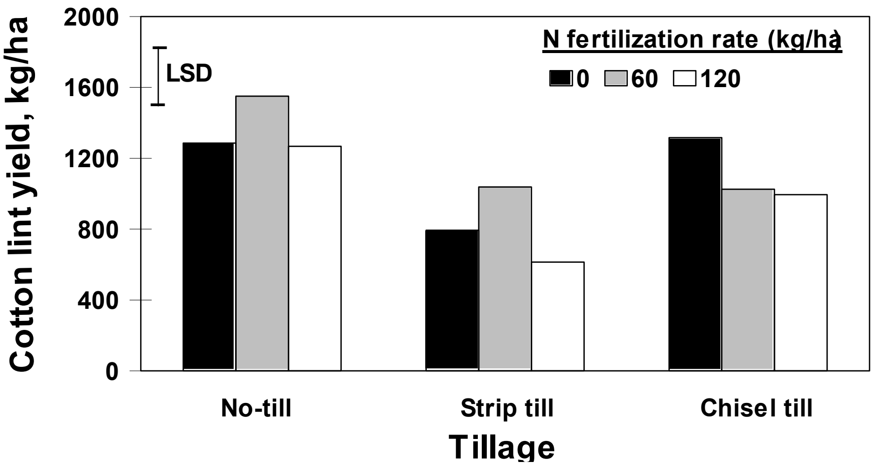

**FIGURE 6.** Cotton lint yield as influenced by tillage and N fertilization rates averaged across cover crops (rye, hairy vetch, rye + hairy vetch mixture and no cover crop) in 2000. LSD denotes least significant difference at $p < 0.05$.

Because increasing C and N storage in the soil increases sequestration of atmospheric C and N, these practices will also help to reduce global warming. As agricultural practices remain a major source of greenhouse gas emissions, improved management practices may restore atmospheric C and N into the soil to their original or higher levels. Further research is necessary to examine if such a combination of management practices can be used for sustaining crop production, conserving soil C and N, reducing N leaching, and helping to reduce global warming.

### ECONOMICAL AND SOCIAL IMPLICATIONS

Before a sustainable management practice can be implemented, it needs to be cost effective. This means that for a farmer or producer to implement such a practice, its economic benefit should outweigh the cost of using it. Although the practice may have many benefits, such as sustaining crop yields, improving soil and water qualities, controlling pests and diseases, or helping to reduce global warming, the economic benefit in the agriculture is usually measured in terms of crop yields while other benefits are often ignored. This is because it is often hard and takes a long time to measure those benefits. In such cases, the benefits averaged across years should be used to calculate the annual return. Other social factors, such as acceptance of the practice to the growers, should also be considered before a practice is fully implemented.

A grower should know all the hidden benefits and costs of a management practice, besides crop production. If the person does not know or understand the benefits, he/she should be fully trained. For example, the total return from crop production system should include return not only from grain production but also from straw production used for animal feed and litter. If the grower decides to incorporate straw into the soil, it may supply C to the soil and enrich soil C storage that may improve soil quality and productivity. In return, a grower may be able to get C credit from the government when C storage in the soil improves.

When a conservation tillage is used, it may save money by reducing the energy required for tillage and the depreciation cost of equipment as compared with those used for conventional tillage. Furthermore, it may increase soil C and N storage by reducing soil erosion and incorporation and decomposition of plant residues. When a cover crop is grown, it may provide many benefits, such as increased soil organic matter concentration, decreased soil erosion and nutrient loss, and lowered incidences of pests, diseases, and weeds. When a legume cover crop is grown, it can reduce the rate of N fertilization by supplying N to the soil from its biomass, thereby reducing N leaching and cost of N fertilization. Similarly, using the reduced rate of N fertilization can decrease the cost of fertilization and potential for N leaching if the reduced rate does not alter crop yield compared with the full rate.

Using sustainable management practices may have some disadvantages, such as an increased cost of production. For example, conservation tillage may need additional use of herbicides to control weeds compared with conventional tillage. Growing cover crops increases costs, such as costs for buying seeds and for the energy required to incorporate cover crop biomass in the soil. Some places may not be suitable for growing cover crops due to harsh winters. In that case, other sustainable management practices may be used and their economic analysis evaluated. The costs for buying seeds for summer crops, fertilizers, pesticides, and equipment use should be taken into account when calculating the cost/benefit ratio. A grower should be able to fully understand all these costs and benefits when the economic analysis of a practice is evaluated.

Janosky et al.[21] reported that conservation tillage produced similar wheat yields and net returns over variable and total costs as conventional tillage. They suggested that no government subsidies are needed for farmers for switching from conventional to conservation tillage system because both tillage systems are equally profitable. As a result, conservation tillage shows great potential for future crop production because it controls soil erosion better than conventional tillage. Similarly, Dhuyvetter et al.[106] found that conservation tillage generally increased net returns when cropping intensity is also increased. While conservation tillage offered additional benefits of improving soil and water qualities, several researchers[66,107,108] reported that total production costs usually increased with the NT production system because of the need of additional herbicides to control weeds. In contrast, Camara et al.[109] found that total production costs for growing spring wheat was lower with NT than with conventional tillage.

Frye et al.[110] observed substantial economic return in corn production using hairy vetch cover crop compared with rye, crimson clover, big flower vetch *(Vicia grandiflora* Koch), or no cover crop in Kentucky. A net return of \$199 $ha^{-1}$ over no cover crop was observed with hairy vetch compared with –\$35 $ha^{-1}$ with rye, \$4 $ha^{-1}$ with crimson clover, and –\$64 $ha^{-1}$ with big flower vetch. When 100 kg $ha^{-1}$ fertilizer N was added, the net return of corn production with cover crop over no cover crop was \$157 $ha^{-1}$ with hairy vetch, \$18 $ha^{-1}$ with rye, –\$6 $ha^{-1}$ with crimson clover, and –\$138 $ha^{-1}$ with big flower vetch. Similarly, Kelly et al.[27] observed a greater economic return in tomato production using hairy vetch cover crop mulch than using polyethylene mulch or bare soil.

Because of the benefits of soil N enrichment by legumes and improving soil and water qualities by nonlegumes, a mixture of legume and nonlegume cover crops may provide the highest economic return. However, due to limited data on the use of a mixed cover crop system on crop production, soil and water qualities, and pest and disease control, more research is needed on the use of such system before a thorough economic analysis is made. Use of such cover crops in a conservation tillage system along with the reduced rate of N fertilization compared with conventional tillage system and recommended rate of N fertilization also needs to be economically evaluated before recommending the practice to the farmers.

### CONCLUSIONS

Improved agricultural management practices, such as conservation tillage, cover cropping, and reduced rate of N fertilization, show promising results to improve soil and water qualities. These practices can improve soil C and N storage and reduce soil erosion and N leaching from the soil profile to the surface and groundwater without significantly altering crop yields as compared with conventional tillage, no cover cropping, and full rate of N fertilization. While conservation tillage can conserve soil organic C and N by reducing oxidation of organic matter and incorporation of plant residues, cover cropping can increase organic C and N concentrations and reduce N leaching by scavenging soil residual N after autumn crop harvest and supplying C and N inputs to the soil from its biomass. If the cover crop is a legume, it can also fix N from the atmosphere and supply greater N input to the soil, thereby reducing the amount and cost of N fertilization. Similarly, a reduced rate of N fertilization can decrease the cost of N fertilization and potential for N leaching. A combination of conservation tillage, mixture of legume and nonlegume cover crops, and reduced rate of N fertilization may show a greater potential for sustaining crop yields and improving soil and water qualities as compared with using either of the practice alone. Because of limited knowledge, further research is needed to examine if such a combination of practices is feasible in various regions and if it is economically and socially acceptable to the growers.

### ACKNOWLEDGMENTS

We appreciate the help of TheScientificWorld Editor for handling the manuscript.

### BIOSKETCHES

**Dr. Upendra M. Sainju** received a B.S. in agriculture from the University of Udaipur, India in 1973, M.S. in soil science from the University of Florida in 1982, and Ph.D. in soil science from the University of Kentucky in 1989. He worked as assistant soil scientist and agronomist from 1973–1980 at the Department of Agriculture in Nepal, as Graduate Research Assistant from 1980–1982 at the University of Florida, as Visiting Professor from 1983–1986 at the Tribhuvan University in Nepal, as Graduate Research Assistant from 1986–1989 at the University of Kentucky, as Postdoctoral Research Associate from 1990–1991 at the Rutgers University in New Jersey, from 1991–1995 at Washington State University, and from 1996 to present at Fort Valley State University in Georgia. He was awarded numerous scholarships for undergraduate and graduate studies. His research interests include soil carbon and nitrogen cycling, carbon and nitrogen sequestrations, nutrient management, soil and water qualities, sustainable crop production, rhizosphere dynamics, and soil and crop management practices. During his professional career, he has authored and co-authored 28 papers in national and international referred journals, 5 papers in symposia proceedings, and 3 papers in book chapters. He has also reviewed more than 10 papers for publication in the referred journals. He brought more than $500,000 grants for research proposals from national and international organizations.

**Dr. Wayne F. Whitehead** received a B.S. in horticulture from Alabama A & M University in 1981, M.S. in plant breeding from Alabama A & M University in 1984, and Ph. D. in plant breeding from the University of Tennessee in 1988. He worked as Hospital Corpsman from 1972–1976 at the U.S. Naval Hospital, as Undergraduate and Graduate Research Assistant from 1978–1983 at Alabama A & M University, as Graduate Research Assistant from 1984–1989 at the University of Tennessee, and as Postdoctoral Research Associate from 1989–1990 at the University of Tennessee, and from 1990 to present in Fort Valley State University in Georgia. He obtained numerous awards for outstanding academic achievements. His research interests include plant nutrition and development of low input management practices for sustainable crop production. He has authored and co-authored 11 papers in journals, proceedings, and bulletins.

**Dr. Bharat P. Singh** received a B.S. in Agriculture from Ranchi University in India in 1962, M.S. in Horticulture from Bhagalpur University in India in 1965, and Ph.D. in Agronomy from Montana State University in 1972. He worked as Agricultural Officer in the State of Bihar, India from 1962–1965, as Graduate Research Assistant at Montana State University from 1969–1972, as Assistant Professor at Fort Valley State University in Georgia from 1973–1978, as Associate Professor in Fort Valley State University in Georgia from 1979–1987, and as Professor at Fort Valley State University in Georgia from 1988 to present. His research interests include sustainable crop production and development of low input management practices. He served as member and chair of various award and working committees in the American Horticultural Society. He authored and co-authored 20 papers in journals, proceedings and bulletins. He brought more than $1 million grant for research proposals from national and international organizations.
